# Evaluating Face2Gene as a Tool to Identify Cornelia de Lange Syndrome by Facial Phenotypes

**DOI:** 10.3390/ijms21031042

**Published:** 2020-02-04

**Authors:** Ana Latorre-Pellicer, Ángela Ascaso, Laura Trujillano, Marta Gil-Salvador, Maria Arnedo, Cristina Lucia-Campos, Rebeca Antoñanzas-Pérez, Iñigo Marcos-Alcalde, Ilaria Parenti, Gloria Bueno-Lozano, Antonio Musio, Beatriz Puisac, Frank J. Kaiser, Feliciano J. Ramos, Paulino Gómez-Puertas, Juan Pié

**Affiliations:** 1 Unit of Clinical Genetics and Functional Genomics, Department of Pharmacology-Physiology, School of Medicine, University of Zaragoza, CIBERER-GCV02 and ISS-Aragon, E-50009 Zaragoza, Spain; alatorre@unizar.es (A.L.-P.); martage.sc@gmail.com (M.G.-S.); marnedo@unizar.es (M.A.); cristinaluca96@hotmail.com (C.L.-C.); rebecantop@gmail.com (R.A.-P.); puisac@unizar.es (B.P.); framos@unizar.es (F.J.R.); 2 Department of Paediatrics, Hospital Clínico Universitario “Lozano Blesa”, E-50009 Zaragoza, Spain; angelaascaso@hotmail.com (Á.A.); lautrujillano@gmail.com (L.T.);; 3 Molecular Modelling Group, Centro de Biología Molecular Severo Ochoa, CBMSO (CSIC-UAM), E-28049 Madrid, Spain; imarcos@cbm.csic.es; 4 Bioscience Research Institute, School of Experimental Sciences, Universidad Francisco de Vitoria, UFV, E-28223 Pozuelo de Alarcón, Spain; 5 Section for Functional Genetics, Institute of Human Genetics, University of Lübeck, 23562 Lübeck, Germany; ilaria.parenti@ist.ac.at (I.P.); frank.kaiser@uk-essen.de (F.J.K.); 6 Institute of Science and Technology (IST) Austria, 3400 Klosterneuburg, Austria; 7 Istituto di Ricerca Genetica e Biomedica, Consiglio Nazionale delle Ricerche, I-56124 Pisa, Italy; antonio.musio@irgb.cnr.it; 8 Institute for Human Genetics, University Hospital Essen, University of Duisburg-Essen, 45147 Essen, Germany

**Keywords:** Cornelia de Lange syndrome, Face2Gene, Facial recognition, Deep learning

## Abstract

Characteristic or classic phenotype of Cornelia de Lange syndrome (CdLS) is associated with a recognisable facial pattern. However, the heterogeneity in causal genes and the presence of overlapping syndromes have made it increasingly difficult to diagnose only by clinical features. DeepGestalt technology, and its app Face2Gene, is having a growing impact on the diagnosis and management of genetic diseases by analysing the features of affected individuals. Here, we performed a phenotypic study on a cohort of 49 individuals harbouring causative variants in known CdLS genes in order to evaluate Face2Gene utility and sensitivity in the clinical diagnosis of CdLS. Based on the profile images of patients, a diagnosis of CdLS was within the top five predicted syndromes for 97.9% of our cases and even listed as first prediction for 83.7%. The age of patients did not seem to affect the prediction accuracy, whereas our results indicate a correlation between the clinical score and affected genes. Furthermore, each gene presents a different pattern recognition that may be used to develop new neural networks with the goal of separating different genetic subtypes in CdLS. Overall, we conclude that computer-assisted image analysis based on deep learning could support the clinical diagnosis of CdLS.

## 1. Introduction

Cornelia de Lange syndrome (CdLS, OMIM #122470, #300590, #610759, #614701, #300882) is a rare congenital condition characterized by a wide spectrum of symptoms and physical features, with a characteristic facial gestalt, intellectual disability, limb reduction and growth retardation as the main phenotypic manifestations [1]. Since symptoms and clinical presentation vary widely in range and severity among affected individuals, clinical diagnosis is often challenging.

CdLS has mainly been associated with genetic variants in genes encoding different structural and regulatory elements of the cohesin complex. In fact, approximately 80% of CdLS patients show pathogenic variants in one of these cohesin complex associated genes. *NIPBL* is the major causative gene and accounts for about 70% of patients with CdLS, while approximately 5%–10% of CdLS patients carry pathogenic variants in *SMC1A*, *SMC3*, *RAD21* or *HDAC8*, *BRD4* and *ANKRD11* genes [2]. Somatic mosaicism in *NIPBL* is frequent in CdLS patients and might further contribute to the different expression of symptoms among patients [3,4,5]. Furthermore, even in the presence of variants affecting the same causative gene, a wide clinical spectrum, from classical to mildly affected patients, is known [6]. The cohesin complex is not only involved in sister chromatids cohesion, but it also exhibits novel biological functions, such as the regulation of gene transcription [7,8,9], thus extending the range of pathomechanisms relevant for cohesinopathies and potentially explaining the variability in the clinical manifestations of CdLS [10,11,12,13]. Furthermore, other chromatin dysregulation disorders [14], cohesinopathies [15] and/or transcriptomopathies [16] present overlapping phenotypes with CdLS, such as CHOPS syndrome (OMIM #616368), KBG syndrome (OMIM #148050), Rubinstein–Taybi syndrome (RSTS, OMIM #180849, #613684), Wiedemann–Steiner syndrome (WDSTS, OMIM #605130), Coffin-Siris syndrome (CSS, OMIM #135900) and Nicolaides–Baraitser syndrome (NCBRS, OMIM #601358).

Deep phenotyping and the standardization of terminologies in the Human Phenotype Ontology have proved to be efficient in the differential diagnosis of many syndromes [17,18]. According to the international consensus statement published in 2018 [2], a classification system based on cardinal and suggestive CdLS features was proposed in order to distinguish between classic and non-classic CdLS phenotypes and to discriminate other entities that resemble CdLS. Four out of six cardinal features are related with facial dysmorphology: (1) Synophrys (HP:0000664) and/or thick eyebrows (HP:0000574); (2) short nose (HP:0003196), concave nasal ridge (HP:0011120) and/or upturned nasal tip (HP:0000463); (3) long (HP:0000343) and/or smooth philtrum (HP:0000319); (4) thin upper lip vermilion (HP:0000219) and/or downturned corners of mouth (HP:0002714). Therefore, evaluation of facial dysmorphism is of outmost importance for the diagnosis of CdLS. However, it largely depends on the expertise of the examining physician.

Within the last years, artificial intelligence (AI) systems have supported several clinical and diagnostic activities, such as visual diagnoses in pathology, radiology, dermatology and ophthalmology. Besides, computational assistance is gaining more attention in the field of medical/clinical genetics, in which deep learning technologies have been increasingly applied to identify facial phenotypes of rare genetic disorders. Very recently, Gurovich et al. [19] proposed a new technology powering Face2Gene, DeepGestalt, which comprises over 17,000 facial images for more than 200 rare diseases, achieving 91% top-10 accuracy.

The aim of this study was to assess the current clinical utility of Face2Gene technology in CdLS diagnosis by testing a cohort of 49 individuals already clinically and molecularly confirmed as CdLS. We explore sensitivity for facial image recognition of CdLS patients using probands of various ages and with causative variants in different causative genes.

## 2. Results

### 2.1. Clinical and Molecular Diagnosis of the Individuals Analysed

According to the clinical score published by Kline et al. 2018, 36 of the 49 patients showed a classic phenotype (score > 11), six showed a non-classic phenotype (score 9–11), and three cases presented a clinical score of 8. For four patients, we were not able to calculate the score due to the lack of some critical information (Table 1).

For all 49 individuals reported, causative genetic variants in known CdLS genes were identified and confirmed. Genetic variants in *NIPBL* were identified in 33 individuals, five patients showed variants in *HDAC8*, eight in *SMC1A* and three in *RAD21*. The 49 individuals harboured 47 unique variants and one recurrent non-frameshift deletion (*SMC1A*, c.802-804delAAG) that was identified in three unrelated patients (Table 1).

Twenty-two of these patients were reported and the remaining 27 were unpublished. Whereas already known genetic variants were identified in 11 of those patients, 16 individuals presented new causal variants that were not previously identified. Eleven of them affected *NIPBL* (two missense variants, three nonsense variants, two splice variants, two frameshift variants and a deletion involving exon 4), two variants affect *SMC1A* (one missense and one splice variant), two missense variants affect *HDAC8* and two were microdeletions, including the *RAD21* gene (Table 1).

For those variants tested, the vast majority (27 of 31) were de novo. Two of the four variants inherited from a clinically diagnosed parent affected *NIPBL*: One *SMC1A* and one *RAD21* (Table 1).

### 2.2. Identifying Cornelia de Lange Syndrome using Face2Gene

**Figure 1:**
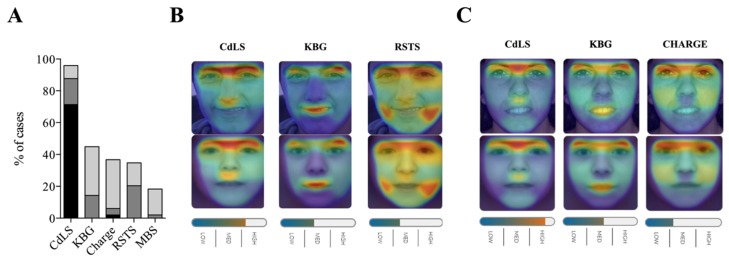
Face2Gene facial analysis in a cohort of 49 patients with CdLS and molecular diagnosis. (**A**) Top-five sensitivity of the five most frequent syndromes listed. High, medium or low gestalt level frequencies are shown. (**B**) Image comparison of a representative case (N05) with a variant in *NIPBL* and the mask syndrome elaborated for CdLS, KBG and Rubinstein–Taybi syndrome (RST), respectively. (**C**) Image comparison of a representative case (N09) with a variant in *NIPBL* gene and the mask syndrome elaborated for CdLS, KBG and Charge syndromes, respectively.

Based on the profile images of the Spanish National CdLS Cohort with causative variants described in *NIPBL*, *SMC1A*, *HDAC8* or *RAD21* genes, CdLS was submitted as one of the top five in the sorted suggestion list of Face2Gene in 47/49 cases (97.9%). Furthermore, CdLS was suggested as the most probable clinical diagnosis in 41/49 cases (83.7%). Among 41 cases in which CdLS was ranked in the top five, 35 cases (71.5%) obtained the high gestalt level. Medium and low gestalt levels were achieved in eight and four cases (16.3% and 8.1%), respectively (Figure 1; Supplementary Table S1).

Interestingly, all 12 cases with nonsense or frameshift variants presented a high gestalt level for CdLS. Furthermore, regarding clinical score, CdLS was proposed as the most probable clinical diagnosis in 32/36 (88.8%) cases with classic phenotype, and 6/9 (66.6%) cases with a clinical score <11 (Table 1 and Supplementary Table S1).

KBG syndrome was the second most suggested diagnosis. In 22/49 (44.89%) cases, it was in the top five list. CHARGE syndrome, Rubinstein–Taybi syndrome and Moebius syndrome were also mentioned in the top five list, with frequencies of 36.7% (18/49), 34.7% (17/49), and 18.4% (9/49), respectively (Figure 1; Supplementary Table S1). In two cases (#N19 *NIPBL* gene; #S36, *SMC1A* gene), KBG syndrome was the first suggested as most the probable clinical diagnosis. In one case, Rubinstein–Taybi syndrome (#S40, *SMC1A* gene) and Charge syndrome were suggested as the first diagnosis for a H46 with a variant in the *HDAC8* gene. None of them presented a high gestalt level.

### 2.3. Face2Gene Evauation for Facial Images of CdLS Patients at Different Ages

No differences in top-one sensitivity were observed between the facial images of the youngest and oldest probands analysed (*n* = 49, from 1- to 41-years-old, median = 5, mean = 10.3) (Supplementary Table S1). However, in order to evaluate if Face2Gene performance may have been affected by the age at which the facial images were taken, we analysed 49 photos from 15 patients at different ages (from 1- to 33- years-old). Regarding top-one result, there was a complete agreement between images of the same patient, even in #S36 and #H46 probands, who showed KBG and Charge syndrome as a first option. Despite this specificity, the top-five diagnosis varied considerably between the different ages, although the second-rank syndrome showed some consistent tendency (Supplementary Table S2).

### 2.4. Face2Gene Evaluation for Facial Image of CdLS Patients with Different Causative Genes

**Figure 2:**
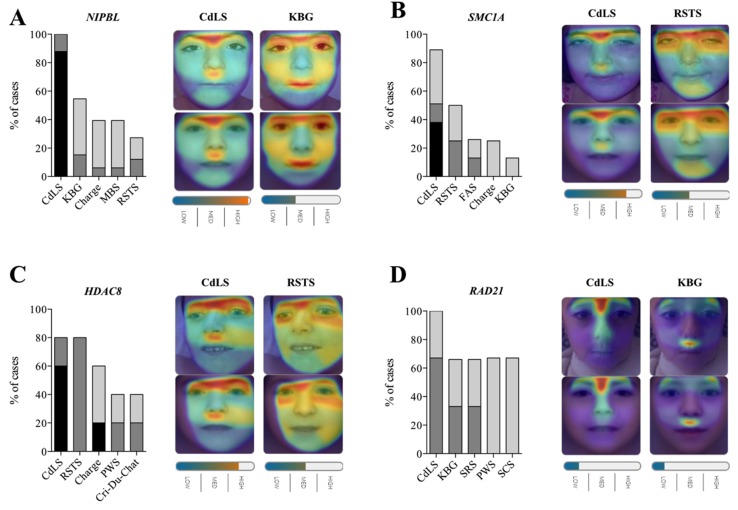
Face2Gene evaluation for facial image of CdLS patients with variants in different causative genes. For each gene, top-five sensitivity of the five most frequent syndromes are listed. High, medium or low gestalt level frequency is indicated. For each gene, a representative image is shown illustrating the mask elaborated for CdLS, KBG or RSTS syndromes (bottom) for the different photographs analysed (top). Overlapping facial regions are indicated by the coloured halo from red to blue. (**A**) *NIPBL* gene (*n* = 33), individual #N08 (**B**) *SMC1A* gene (*n* = 8), individual #S37. (**C**) *HDAC8* gene (*n* = 5), individual #H43. (**D**) *RAD21* gen (*n* = 3), individual #R49. CdLS, Cornelia de Lange syndrome; FAS, Fetal alcohol syndrome; KBG, KBG syndrome; MBS, Moebius syndrome; PWS, Prader–Willi syndrome; RSTS, Rubinstein–Taybi syndrome; SRS, Silver–Russell syndrome; SCS, Saethre–Chotzen syndrome.

Next, we examined whether Face2Gene was able to discriminate facial phenotypes of CdLS patients depending on the genetic variants in different genes. The top-five sensitivity for the *NIPBL, HDAC8, SMC1A* and *RAD21* clinical test set led to a similar result with little or no variation: 100% (33/33), 80% (4/5), 87.5% (7/8) and 100% (3/3), respectively. However, the top-one sensitivity presented more differences between genotypes: 97.0% (32/33), 60% (3/5), 50.0% (4/8), and 66.6 % (2/3), respectively. A high gestalt level was obtained in 87.9% (29/33), 60.0% (3/5), 37.5% (3/8) and 0.0% (0/3) of patients with causative variants described in *NIPBL*, *HDAC8*, *SMC1A* and *RAD21* genes, respectively (Figure 2) (Supplementary Table S1).

Interestingly, the differential diagnosis seemed to be correlated with the affected gene. Whereas for *NIPBL* and *RAD21* patients, KBG syndrome was the second most common diagnosis (top-five rank), 54.5% (18/33) and 66,7% (2/3), Rubinstein–Taybi syndrome appeared most frequently in *SMC1A* and *HDAC8* patients, 50.0% (4/8) and 80.0% (4/5), respectively (Figure 2) (Supplementary Table S1).

**Figure 3:**
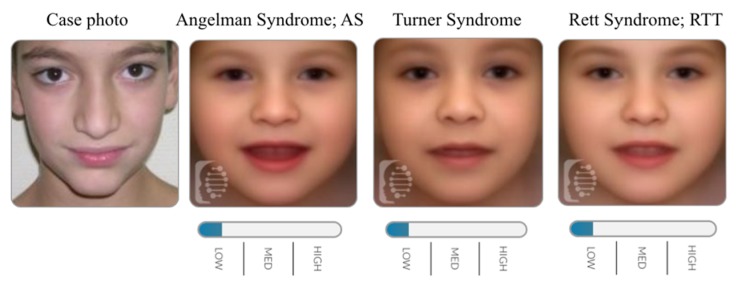
Face2Gene evaluation for facial image of a patient harbouring a duplication segment encompassing *SMC1A* gene.

At the same time, we analysed a special case, which was previously published with a duplication involving the *SMC1A* gene [30]. As described, the patient showed a classical CdLS facial gestalt. Indeed, CdLS diagnosis did not appear between the 30 diagnoses provided by Face2Gene. This allowed us to strengthen that *SMC1A* duplication acts as a cohesinopathy, but not as CdLS, at least in terms of facial features (Figure 3).

## 3. Discussion

Deep learning is an exciting and promising approach that has already been successfully established in various clinical fields and is now gaining attention in clinical genetics. It consists of a variation of machine learning that uses neural networks to automatically extract novel features from input data [31]. In this respect, new technologies, such as a smartphone app called Face2Gene, have been recently developed [19]. Face2Gene uses deep-learning algorithms to help genetic clinicians and paediatricians diagnose conditions by image recognition.

This is of particular interest for CdLS, in which heterogeneity in clinical presentation and phenotype overlap with different syndromes, and the still widely unknown molecular pathomechanisms makes its diagnosis challenging for paediatricians. Thus, we have evaluated Face2Gene usefulness and sensitivity in a large cohort consisting of 49 patients with CdLS syndrome molecularly confirmed by mutations in *NIPBL*, *SMC1A*, *HDAC8* and *RAD21* genes.

Face2Gene technology has proved to have a high sensitivity detecting CdLS. In our cohort, we obtained a 97.9% top-five sensitivity, which is even higher than the 95.97% overall top-ten sensitivity rate reported by Gurovich et al. in a cohort of CdLS patients [19], and considerably higher than other previous applications [32,33]. Since Face2Gene was built as a framework that learns from every solved case, it can be expected that sensitivity will be even improved. However, we are aware that this high sensitivity might decrease precision, and specificity studies are required in order to further evaluate accuracy and clinical usage of Face2Gene.

The ages at which the photographs were taken were reported to affect Face2Gene results, at least in patients with inborn errors of metabolism [34] and in cases with Down syndrome [35]. Additionally, it has been proposed that the changing facial features of CdLS over time, specifically the coarsening of the eyebrows and eyes and prognathism, may increase the difficulty of a diagnosis in older individuals [32]. Despite that, we did not find substantial differences in sensitivity regarding the age at which CdLS facial images were taken.

Our results suggest differences in sensitivity depending on the clinical score and affected gene. The study shows that Face2Gene was able to diagnose CdLS patients with classic phenotype (clinical score >11) with a top-one sensitivity of 88.8%. However only 66.6% top-one sensitivity was achieved in non-classic phenotypes (clinical score < 11). When evaluating within genetic groups, *NIPBL* patients obtained the maximum sensitivity with 97% top-one sensitivity, which makes sense since individuals with the classic CdLS phenotype are more likely to harbour variants in *NIPBL* gene [2].

Finally, although this study had limitations in terms of the sample size of some genotypes, it is remarkable that each gene presented a different pattern recognition. We found that KBG syndrome was the second diagnosis for individuals harbouring causative variants in *NIPBL* and *RAD21* genes. However, in *SMC1A* patients, it was the fifth in the list, while in *HDCA8*, it did not even appear in the top five. Nevertheless, in patients with *SMC1A* and *HDAC8* variants, Rubinstein–Taybi was the second most common diagnosis, being the fifth in the list of *NIPBL* and insistent in the lists of *RAD21* individuals. Furthermore, regarding gestalt score, it is noticeable that *NIPBL* shows pronounced facial features, whereas these are softened in *RAD21* patients. This dramatically reduces the ability of Face2Gene to diagnose patients with alterations in *RAD21* gene. Taking into account that *RAD21* is an essential part of the cohesin ring structure, as well as *SMC1A* and *SMC3*, this difference is not easy to explain from a molecular point of view. Perhaps it is related to the less central role of *RAD21* in the ATPase function of the other two proteins [36], but this is an extreme that should be studied in more detail in the future.

Nevertheless, as it has been shown in Noonan syndrome [19], the differences in pattern recognition between genes involved in CdLS will be useful for training and refining neural networks to be able to discriminate between genetic subtypes in CdLS. For example, it has been described that thicker eyebrows are suggestive of a mutation in *SMC1A* or *SMC3* [32], and females containing variants in *HDAC8* tend to present hypertelorism and a slightly bulbous nasal tip [12] All these data point out that Face2Gene can be optimized for specific CdLS phenotypic subsets if the number of photographs of different genotypes increases considerably.

In conclusion, the application of deep learning to image recognition is an exciting area that is developing rapidly and is primed to revolutionize clinical genetics diagnosis. The potential to offer the ability to analyse facial image at a speed and sensitivity never seen before is giving physicians a new tool in CdLS diagnosis management. Moreover, future studies based on deep learning will allow us to account for genotype–phenotype correlations between mutation type and affected gene in CdLS. There is still much work to be done to fully understand the correlations between genotype and phenotype in individuals with CdLS and CdLS-like diagnoses, but there are fascinating emerging applications with increasingly accurate use by clinicians.

## 4. Materials and Methods

Participants: We compiled a cohort comprising 49 (30 females and 19 males) individuals with a clinical diagnosis of CdLS. We limited our analysis to patients with both molecular diagnosis and available frontal facial photographs. Most of the patients were referred by the Spanish Association of Cornelia de Lange Syndrome (CdLS Spain). Written informed consent from parents or guardians was obtained. The protocol study was approved by the Ethics Committee of Clinical Research from the Government of Aragón (CEICA; PI16/225). Only individuals that were consented for publication of facial photographs were shown in this study.

Clinical Score: The CdLS clinical score was computed by trained physicians according to the international consensus criteria of CdLS [2]. A combination of cardinal and suggestive features was used to diagnose the CdLS phenotype. Classic CdLS was indicated with a score of > 11 if at least three cardinal features were identified, while a score of 9–10 indicated non-classic CdLS and implied the presence of at least two cardinal features.

Molecular Diagnosis: All individuals with CdLS were subjected to molecular analysis by next generation sequencing. In the majority of cases, targeted gene-panel via deep sequencing analysis was performed using Ion Chef and Ion S5 XL Systems (Thermo Fisher Scientific). The custom CdLS panel was designed using the Ion AmpliSeq^TM^ Designer online tool. The designed panel spanned 249.25 kb of the selected genomic sequencing, including *NIPBL* (NM_133433.3), *SMC1A* (NM_006306.3), *SMC3* (NM_058243.2), *RAD21* (NM_006265.2), *HDAC8* (NM_018486.2), *BRD4* (NM_058243.2) and *ANKRD11* (NM_001256183.1). The analysis was performed using Ion Reporter and IGV (Broad Institute) software. Reportable variants were validated by Sanger sequencing. If the panel did not detect causal variants, multiplex ligation-dependent probe amplification (MLPA) and/or comparative genomic hybridisation (CGH) array were done. Human Genome Variation Society (www.hgvs.org) nomenclature guidelines were used to name the mutation at the DNA level and the predicted resulting protein.

Facial and Data Analysis: We analysed the frontal images of all individuals using Face2Gene technology (FDNA Inc., Boston, MA, USA; https://www.face2gene.com). According to Gurovich et al. [19], we evaluated the sensitivity of Face2Gene by measuring the top-one and top-five sensitivity. Thus, top-one sensitivity means that the correctly diagnosis syndrome was suggested the first in the sorted suggestion list of Face2Gene, while top-five sensitivity means that it was suggested as one of the top five on that list. Furthermore, for each analysed individual, we evaluated the gestalt similarity using the “gestalt level” barplot, which indicates levels of “high,” “medium,” and “low.”

Statistical analyses and graphics were produced with GraphPad Prism 6 software.

## Figures and Tables

**Table 1:** Population description, clinical score and potential causative variants.

ID	Sex	Age Photo	CdLS Score	Gene	Variant Type	Exon/Intron	Mutation (hg 19)	Protein	Inheritance	Novelty
#N01	M	5	14	*NIPBL*	missense	35	c.6242G>T	p.Gly2081Val	de novo	Patient reported [20]
#N02	M	1	10	*NIPBL*	splice variant	3i	c.230+1G>A	-	de novo	Patient reported [20]
#N03	F	2	13	*NIPBL*	nonsense	10	c.2146C>T	p.Gln716*	de novo	Patient reported [20]
#N04	F	1	15	*NIPBL*	missense	37	c.6449T>C	p.Leu2150Pro	-	Patient Reported [2,20]
#N05	M	14	12	*NIPBL*	nonframeshiftDeletion	30	c.5689_5691delAAT	p.Asn1897del	de novo	Patient Reported [2,20]
#N06	F	4	13	*NIPBL*	frameshiftDeletion	20	c.4321G>T	p.Phe1442Lysfs*3	de novo	Patient reported [20]
#N07	F	13	16	*NIPBL*	frameshiftDeletion	10	c.2479_2480delAG	p.Arg827Glyfs*2	de novo	Patient reported [20]
#N08	M	9	14	*NIPBL*	nonsense	3	c.133C>T	p.Arg45*	de novo	New CdLS Variant
#N09	F	24	9	*NIPBL*	missense	36	c.6316G>C	p.Val2106Leu	de novo	ClinVar
#N10	F	32	15	*NIPBL*	missense	41	c.7012G>C	p.Ala2338Pro	de novo	ClinVar
#N11	F	37	15	*NIPBL*	splice variant	32i	c.5862+2insGAG	-	de novo	Similar Variant described in the literature c.5862 + 1delG [21]
#N12	M	16	15	*NIPBL*	frameshiftDeletion	10	c.3060_3063delAGAG	p.Glu1021Thrfs*22	-	Variant described in the literature [11]
#N13	F	27	13	*NIPBL*	missense	29	c.5471C>T	p.Ser1824Leu	de novo	New CdLS Variant
#N14	F	40	-	*NIPBL*	missense	40	c.6893G>A	p.Arg2298His	de novo	Variant described in the literature [11,22,23]
#N15	M	13	10	*NIPBL*	missense	47	c.8387A>G	p.Tyr2796Cys	familial (m)	Patient reported [24]
#N16	F	2	14	*NIPBL*	missense	36	c.6269G>T	p.Ser2090Ile	de novo	Patient reported [20]
#N17	M	3	13	*NIPBL*	splice variant	28i	c.5329-6T>G	-	familial (p)	Patient reported [25]
#N18	F	8	13	*NIPBL*	frameshiftDeletion	44	c.7438_7439delAG	p.Arg2480Lysfs*5	de novo	Patient reported [20]
#N19	M	16	13	*NIPBL*	exon 4 deletion	4	-	-	-	New CdLS Variant
#N20	F	5	15	*NIPBL*	missense	39	c.6647A>C	p.Tyr2216Ser	de novo	Patient reported [4]
#N21	F	7	14	*NIPBL*	missense	36	c.6272G>A	p.Cys2091Tyr	-	Variant described in the literature [26]
#N22	M	1	16	*NIPBL*	splice variant	19i	c.4320+5G>C	-	de novo	Patient reported [20,25]
#N23	F	5	14	*NIPBL*	nonsense	39	c.6880C>T	p.Gln2294*	de novo	Patient reported [20]
#N24	F	1	15	*NIPBL*	nonsense	9	c.1445_1448delGAGA	p.Arg482Asnfs*20	-	Patient reported [27]
#N25	M	3	16	*NIPBL*	missense	39	c.6647A>G	p.Tyr2216Cys	de novo	Patient reported [28]
#N26	F	7	13	*NIPBL*	missense	40	c.6860T>C	p.Leu2287Pro	-	New CdLS Variant
#N27	M	1	15	*NIPBL*	nonsense	29	c.5455C>T	p.Arg1819*	de novo	New CdLS Variant
#N28	F	9	15	*NIPBL*	frameshiftInsertion	41	c.6964_6965insATTTA	p.Ala2325*	-	New CdLS Variant
#N29	F	2	13	*NIPBL*	splice variant	21i	c.4560+4A>G	-	de novo	New CdLS Variant
#N30	F	1	15	*NIPBL*	frameshiftDeletion	38	c.6549_6552delCTCA	p.His2183Glnfs*13	de novo	New CdLS Variant
#N31	M	4	17	*NIPBL*	splice variant	20i	c.4422-1G>T	-	-	New CdLS Variant
#N32	M	34	14	*NIPBL*	splice variant	2i	c.65-5A>G	-	-	LOVD
#N33	F	16	15	*NIPBL*	nonsense	9	c.992C>T	p.Arg308*	-	New CdLS Variant
#S34	M	5	12	*SMC1A*	missense	4	c.587G>A	p.Arg196His	de novo	Patient reported [20,29]
#S35	F	27	14	*SMC1A*	nonframeshiftInsertion	5	c.802_804delAAG	p.Lys268del	de novo	Patient reported [20]
#S36	M	4	13	*SMC1A*	missense	13	c.2132 G>A	p.Arg711Gln	de novo	Patient reported [20]
#S37	F	7	14	*SMC1A*	missense	15	c.2369G>A	p.Arg790Gln	-	Patient reported [13]
#S38	F	2	-	*SMC1A*	nonframeshiftDeletion	5	c.802_804delAAG	p.Lys268del	-	Variant described in the literature [20]
#S39	F	2	13	*SMC1A*	splice variant	2	c.44-1G>A	-	-	New CdLS Variant
#S40	M	11	-	*SMC1A*	missense	22	c.3340A>T	p.Asn1114Tyr	familial (m)	New CdLS Variant
#S41	F	41	15	*SMC1A*	nonframeshiftDeletion	5	c.802_804delAAG	p.Lys268del	-	Variant described in the literature [20]
#H42	F	4	8	*HDAC8*	missense	6	c.562G>A	p.Ala188Thr	de novo	Clin Var
#H43	M	3	12	*HDAC8*	missense	9	c.958G>A	p.Gly320Arg	-	ClinVar
#H44	F	6	9	*HDAC8*	missense	7	c.709G>T	p.Asp237Tyr	-	New CdLS Variant
#H45	M	5	11	*HDAC8*	missense	4	c.305G>A	p.Cys102Tyr	de novo	New CdLS Variant
#H46	F	11	8	*HDAC8*	missense	5	c.468T>G	p.Asn156Lys	de novo	Patient reported [12]
#R47	F	3	8	*RAD21*	missense	11	c.1382C>T	p.Thr461Ile	familial (p)	Patient reported (In press)
#R48	F	5	-	*RAD21*	4.7 Mb deletion	whole gene	8q24.11q24.12(117765326_122494596)x1		-	New CdLS Variant
#R49	M	8	10	*RAD21*	504 Kb deletion	whole gene	8q24.11 (117765326_118270323)x1		-	New CdLS Variant

Abbreviations: M, male; F, female; (m), maternal; (p), paternal.

## Supplementary Materials

Supplementary materials can be found at https://www.mdpi.com/1422-0067/21/3/1042/s1.

## Abbreviations

CdLS	Cornelia de Lange Syndrome
RSTS	Rubinstein-Taybi Syndrome
PMID	PubMed Unique Identifier
