# Supplementary material for: Evaluating Face2Gene as a Tool to Identify Cornelia de Lange Syndrome by Facial Phenotypes
Source: Int J Mol Sci. 2020 Feb 4;21(3):1042. doi: 10.3390/ijms21031042 (PMC7038094; doi:10.3390/ijms21031042)
Supplement: Supplementary file 1 [file ijms-21-01042-s001.pdf]

Supplementary Table 1: Face2Gene facial analysis. The most frequent syndromes are listed. The value in the cells is the position in the sorted suggestion list. Shades of black indicate high gestalt level, shades of gray indicate mild gestalt level and shades of pale gray indicate low gestalt level.

ID	Gen	Age	CdLS	KBGS	RSTS	CHARGE	MBS	ML4	FAS	BRWS	CSS	EDSMC1	HFM	Turner	Cri-Du-Chat	SBBYSS	RTT	PWS
#N01	NIPBL	5	1						5		1							
#N02	NIPBL	1	1															
#N03	NIPBL	2	1		3							4						
#N04	NIPBL	1	1		3	5												
#N05	NIPBL	14	1	2	3									5				
#N06	NIPBL	4	1	3			2	4										
#N07	NIPBL	13	1	5				2			4	3						
#N08	NIPBL	9	1	2			4						5					
#N09	NIPBL	24	1	2		3					4							
#N10	NIPBL	32	1	3									5					
#N11	NIPBL	37	1		3			2				4						
#N12	NIPBL	16	1		5	3	4	2										
#N13	NIPBL	27	1					2									3	
#N14	NIPBL	40	1	4	3													
#N15	NIPBL	13	1	5									4		3			
#N16	NIPBL	2	1	2		4	3					5						
#N17	NIPBL	3	1	3					4						5			
#N18	NIPBL	8	1			3				2								
#N19	NIPBL	16	2	1					3									
#N20	NIPBL	5	1	3		4	2											
#N21	NIPBL	7	1			4				5								
#N22	NIPBL	1	1	2			4			5								
#N23	NIPBL	5	1	3		4												
#N24	NIPBL	1	1		3	2												
#N25	NIPBL	3	1	2										4				
#N26	NIPBL	7	1	5			2	3					4					
#N27	NIPBL	1	1	5	3					2								
#N28	NIPBL	9	1				2		3									
#N29	NIPBL	2	1			2												4
#N30	NIPBL	1	1	4	2	5												
#N31	NIPBL	4	1			4												
#N32	NIPBL	34	1															
#N33	NIPBL	16	1			5												
#S34	SMC1A	5	1					5	2						3			
#S35	SMC1A	27	1															
#S36	SMC1A	4	2	1					4									
#S37	SMC1A	7	1		2												4	
#S38	SMC1A	2	3		2													
#S39	SMC1A	2	3		1	4												
#S40	SMC1A	11	1															
#S41	SMC1A	41			4	1												
#H42	HDAC8	4	1	5	2								4		3			
#H43	HDAC8	3	1		2					5					4			
#H44	HDAC8	6				4	5									1	2	
#H45	HDAC8	5	1		2	4												5
#H46	HDAC8	14	3		4	1												2
#R47	RAD21	3	2	3														5
#R48	RAD21	5	1								3							
#R49	RAD21	8	1	3														5

Abbreviations: BRWS, Baraitser-Winter Syndrome; CdLS, Cornelia De Lange Syndrome; CSS, Coffin-Siris Syndrome; EDSMC1, Ehlers-danlos syndrome musculocontractural type 1; FAS, Fetal Alcohol Syndrome; HFM, Hemifacial Microsomia; KBG, KBG Syndrome; MBS, Moebius Syndrome; ML4, Mucolipidosis Type IV; RTT, Rett Syndrome; RSTS, Rubinstein-Taybi Syndrome; SBBYSS, Ohdo Syndrome, SBBYS Variant.

Supplementary Table2: Face2Gene facial analysis of 16 patients with CdLS at different ages. For each case the most frequent syndromes are listed. The value in the cells is the position in the sorted suggestion list. Shades of black indicate high gestalt level, shades of gray indicate mild gestalt level and shades of pale gray indicate low gestalt level.

ID	Age	CdLS	FAS	SLOS	MBS	KBGS	BRWS	CSS	Cri-Du-Chat	FVS	RSTS
**#N01**	5	1		4			2	3		5	
	11	1	3	2	5	4					
	13	1	3		2				4		5

ID	Age	CdLS	KBGS	HFA	Turner	RSTS	Stickler	Sotos	MBS	SLOS
**#N05**	6	1	2				3		4	5
	14	1	2	4	5	3				
	15	1	2	5	4			3		

ID	Age	CHARGE	PWS	USP7-Related Disorder	CdLS	HFM	RSTS	AS	CSS	RTT	KLEFS	22q11.2 Deletion
**#H46**	9	1	3	2				4			5	
	11	1	2		3		4					5
	14	1	2			3			4	5		

ID	Age	CdLS	KBGS	GLHS	MNKES	LMNS	MBS	ML4	FAS	BWS	CLS
**#N08**	1	1		3		2		4		5	
	6	1	2	3	5				4		
	9	1	2		4		3				5

ID	Age	CdLS	MBS	CHARGE	MRX102	ML4	KBGS	FAS	GLHS	AS
**#N28**	9	1	2	3	5			4		
	12	1	2		5		3		4	
	13	1	2	4		3				5

ID	Age	KBGS	CdLS	Glass	AAS	PHMDS	FAS	MRX102	CLS	SMS	DM
**#S36**	1	1	2		3			4		5	
	3	1	2	3					4		5
	4	1	2	5		3	4				

ID	Age	CdLS	KBGS	CHARGE	CSS	HFM	MRX102	MFS	RSTS	FVS	HFA
**#N09**	21	1	2	3	4	5					
	24	1	2	3	5		4				
	26	1	2			3			4		5
	33	1			2		5	3		4	

ID	Age	CdLS	ML4	RTT	Sotos	MNKES	MRXSL	PWS	KBGS	HFA	BCNS	MRX102	BBS	JBTS
**#N13**	4	1	5			2		3		4				
	10	1	2				3		4					5
	17	1		2	3						4		5	
	27	1	2	3	4							5		

ID	Age	CdLS	SGBS1	KBGS	MBS	RSTS	CHARGE	BWS	CSS	HFM	Campomelic Dysplasia	PKS
#N21	2	1	4		3	2				5		
	3	1	2	4				3			5	
	5	1	3	2					4			5
	7	1	2	4	3		5					

ID	Age	CdLS	RTT	RSTS	Sotos	MCAP	PYCR2 Mutations	MBS	ML4	SMS	MNKES	HPMRS	WDSTS
#S37	2	1	3				2			4		5	
	3	1	4	5	3	2							
	4	1	2					3	4				5
	7	1	4	2	3						5		

ID	Age	CdLS	KBGS	CHARGE	MBS	EDSMC1	RSTS	Waardenbur	BRWS	BOPS	MCAP	HPMRS
#N16	1	1		4		5		2			3	
	2	1	2	4	3	5						
	3	1	2		4				3			5
	5	1	5	4			2			3		

ID	Age	CdLS	KBGS	RSTS	CHARGE	ML4	HFM	LIS1	MBS	CSS	MPS
#N26	1	1	5	4	3			2			
	2	1	2	4	3						5
	3	1		3		2	5			4	
	7	1	5			3	4		2		

ID	Age	CdLS	MBS	KBGS	CHARGE	HFM	ML4	MDLS	WDSTS
#N20	2	1	3	2		4			5
	5	1	2		3		4	5	
	6	1	2	3	4	5			

ID	Age	CdLS	MBS	CHARGE	UFS	KBGS	BBS	EDSMC1	PWS
#N07	4	1	3	2			4		5
	13	1	5		2	3		4	

ID	Age	CdLS	RSTS	Cri-Du-Chat	Noonan	CHARGE	CFSMR	LDS	BRWS
#N12	5	1	2		3	4		5	
	16	1	2	3			4		5

Abbreviations: AS, Angelman syndrome; ASS, Aarskog-Scott syndrome; BBS, Bardet-Biedl Syndrome; BCNS, Basal cell nevus syndrome; BOPS, Bohring-Opitz syndrome; BRWS, Baraitser-Winter Syndrome; CdLS, Cornelia De Lange Syndrome; CFSMR, Craniofacial dysmorphism, skeletal anomalies, and mental retardation syndrome; CSS, Coffin-Siris Syndrome; DM, Myotonic Dystrophy; EDSMC1, Ehlers-danlos syndrome musculocontractural type 1; FAS, Fetal Alcohol Syndrome; FVS, Fetal Valproate Syndrome; GLHS, Gomez-Lopez-Hernandez syndrome HFA, Hemifacial atrophy, progressive; HFM, Hemifacial Microsomia; HPMRS, Hyperphosphatasia with mental retardation syndrome 1; JBTS, Joubert Syndrome; KBG, KBG Syndrome; KLEFS, Kleefstra Syndrome; LDS, Loeys-Dietz Syndrome; MBS, Moebius Syndrome; MCAP, Megalencephaly-capillary malformation-polymicrogyria syndrome; MFS, Marfan Syndrome; ML4, Mucolipidosis Type IV; MPS, Mucopolysaccharidoses; MNKES, Muenke syndrome; MRXSL, Mental retardation, X-linked syndromic, Lubs type; MRX102, Mental retardation, X-linked 102; PHMDS, Phelan-McDermid syndrome; PKS, Pallister-Killian syndrome; PWS, Prader-Willi Syndrome; RSTS, Rubinstein-Taybi Syndrome; RTT: Rett Syndrome; SBBYSS, Ohdo Syndrome, SBBYS Variant; SLOS, Smith-Lemli-Opitz syndrome; SMS, Smith-Magenis syndrome; UFS, Urofacial Syndrome; WDSTS, Wiedemann-Steiner syndrome.
